# Targeting Cytokines and Their Pathways for the Treatment of Cancer

**DOI:** 10.3390/cancers15215224

**Published:** 2023-10-30

**Authors:** Amy B. Heimberger, Shashwat Tripathi, Leonidas C. Platanias

**Affiliations:** 1 Department of Neurological Surgery, Feinberg School of Medicine, Chicago, IL 60611, USA; shashwat.tripathi@northwestern.edu; 2 Robert H. Lurie Comprehensive Cancer Center of Northwestern University, Chicago, IL 60611, USA; l-platanias@northwestern.edu; 3 Division of Hematology Oncology, Department of Medicine, Feinberg School of Medicine, Chicago, IL 60611, USA; 4 Department of Medicine, Jesse Brown VA Medical Center, Chicago, IL 60612, USA

## 1. Introduction

This Special Issue focuses on the evolving role of immune modulatory cytokines, from their initial use as monotherapeutic recombinant proteins to their more contemporaneous use as modifiers for adoptive cellular immunotherapy. This Special Issue explores sustained delivery platforms such as viral therapy into the tumor microenvironment (TME) that may provide more reasonable clinical delivery schedules while minimizing systemic toxicities. Notably, cytokines have more recently been repositioned to optimize the activation state, effector functions, and persistence of cellular therapeutic products. The discovery of new cytokines at the convergence of immune modulation and tumorigenesis will likely serve as the next generation of cytokine therapeutics. Approaches aimed at targeting cytokine-inducible genes and their protein products are also discussed.

Cytokines serve to modulate inflammation but can also play a pivotal role in either the induction or progression of cancers. Cytokines can be elevated in patients harboring a malignancy, can interact with tumor cells within the TME, or can modulate responses to immune therapeutics. The therapeutic use of cytokines in cancer was likely first exploited in the use of transfer factor. In that case, low-molecular-weight components obtained from Hodgkin’s disease patients in remission were transferred to patients with active disease and stimulated delayed hypersensitivity tests [1]. This undefined product was superseded by the first generation of lymphocyte-elaborated chemokines such as IL-2, IL-4, IL-6, GM-CSF, and IFN-γ characterized and defined by the emerging field of immunology [2]. Based on a PubMed search using the keywords “cytokines” and “cancer” and refined on clinical trials, the use of cytokines in this context reached a zenith in the mid-1990s, but this has subsequently decreased due to a variety of factors, especially off-target toxicities. 

Cytokines can have both tumor cytotoxic and tumor-promoting roles while also mediating either pro-inflammatory or immunosuppressive roles. For example, interleukin 6 (IL-6) has tumor-promoting properties in various cancers, such as lung, breast, and colorectal cancer typically through the triggering of the tumor-promoting and immunosuppressive hub of the signal transducer and activator of transcription (STAT) [3,4,5,6]. In contrast, IL-2 and IL-12 possess pro-inflammatory anti-cancer effects and gene therapy strategies have demonstrated signals of therapeutic response in lung cancer and glioblastoma patients, respectively [7,8]. Some cytokines such as tumor necrosis factor alpha (TNF-α) can have both pro-inflammatory, anti-tumor effects, and tumor-promoting effects, including angiogenesis, epithelial–mesenchymal transition (EMT), or the promotion of tumor proliferation, depending on the concentration [9,10,11,12]. Similarly, transforming growth factor beta (TGF-β) possesses a wide range of activities on tumor progression [13,14].

## 2. Pan-Cancer Heterogeneity of Cytokines

**Figure 1:**
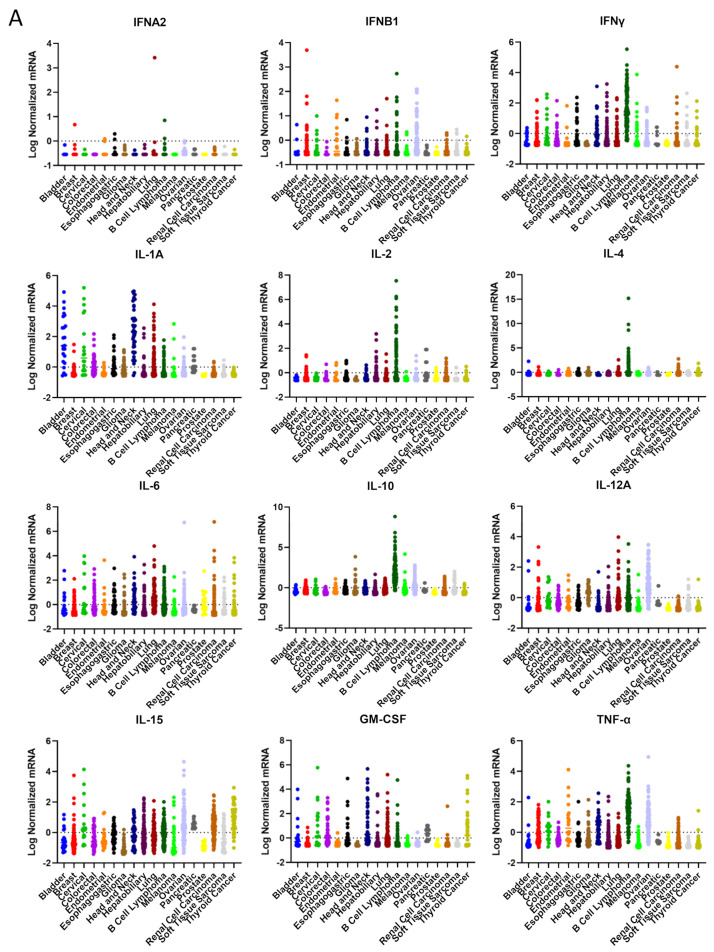

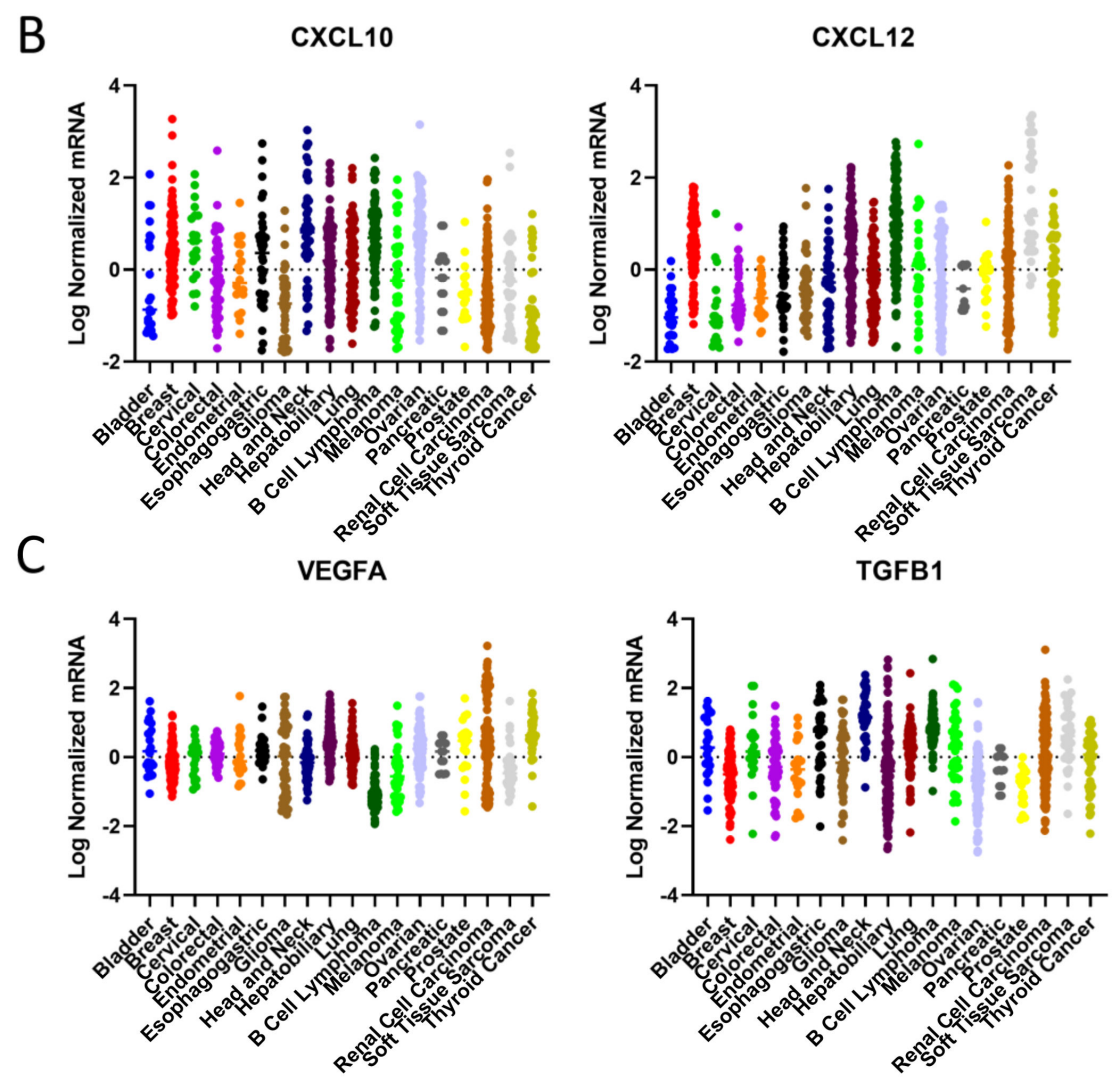
Pan-cancer profiling based on the Cancer Genome Atlas Databases of (**A**) immune cytokines; (**B**) chemokines; (**C**) immunosuppressive mediators. While most cytokines exert pro-inflammatory anti-tumor immune responses, some such as IL-6, TNF-α, and IL-10 have immunosuppressive roles.

Using available cancer atlas datasets, we have interrogated cytokine expression based on mRNA levels to ascertain if there are cancer lineage differences. B cell lymphoma expresses the highest levels and widest variety of cytokines such as IL-1A, IL-2, IL-4, IL-6, IL-10, IL-15, CXCL10, CXCL12, IFNγ, TNF-α, and TGFβ1 (Figure 1). On the opposite end of the spectrum resides pancreatic cancer that is typically devoid of cytokines in general. Most cancers have low levels of IL-2, meaning many patients could theoretically benefit from increasing the levels of this pro-inflammatory cytokine in the TME. Strategies that increase the immune chemokines CXCL10 and CXCL12 within the TME may benefit those with gliomas, as well as endometrial, pancreatic, and prostate cancer patients. In contrast, strategies that eliminate immunosuppressive IL-10 may only be meaningful in cases of gliomas, lymphoma, melanoma, and ovarian cancer. Given the heterogeneity of TGF-β expression, the development of a companion biomarker could be considered for stratification purposes. In the future, it would be interesting to ascertain the profiles of cytokines based on driver mutations as opposed to cancer lineages, which may provide a CLIA-approved enrichment strategy for the identification of appropriate populations. 

## 3. Clinical Studies of Cytokine Gene Therapy

To date, the most extensive clinical efforts for the modulation of cytokines have involved gene therapy strategies mostly using adenovirus as the transfer vector expressing either TNF-α, IL-2, IL-12, IFN-a2b, IFN-β, or GM-CSF in a variety of cancer indications. In a study reported in 1999, a small number of melanoma patients were treated intratumorally with a GM-CSF-encoding vaccinia virus for six weeks. Only mild adverse events were observed, and one patient had a partial response [15]. Later studies used an oncolytic virus genetically modified to express GM-CSF injected into the tumors of patients with melanoma or head and neck, breast, or gastrointestinal cancers that induced anti-tumoral immune response and tumor necrosis in more than half of the subjects [16]. In the subsequent phase II study, 50 patients with unresectable metastatic melanoma were treated with an overall response rate of 26% and a two-year survival rate of 52% [17]. Ultimately, the phase III trial of 436 melanoma patients showed a median survival of 23.3 months relative to the control of tumor-injected GM-CSF protein of 18.9 months [18,19].

Interferons (IFNs) can activate the granzyme B pathway in T cells to inhibit tumor progression [20]. In a phase II study of 43 patients with bladder cancer treated intravesically with a recombinant adenovirus modified to express IFN-α2b, 35% of the patients achieved recurrence-free survival, suggesting that this strategy could be a promising replacement for patients unwilling or unable to undergo radical cystectomy [21]. In a phase I study of malignant mesothelioma, 40 patients treated with the IFN-α2b-expressing adenovirus in combination with chemotherapies had an overall response rate of 25% and a disease control rate of 88%, suggesting a response relative to historical cohorts [22]. In a phase I study of IFN-β genes carried by liposomes injected into patients with high-grade glioma, there was induction antitumor immune responses and tumor infiltrations of macrophages and CD8+ lymphocytes [23]. IFN-α has been used either as monotherapy or with chemotherapy. In the first phase I and II trials of IFN-α, the overall response rate was 16% [24,25,26]. Many of the studies demonstrating the activity of IFN-α were in the adjuvant setting, which ultimately led to FDA approval in 1995 in the indication of melanoma. However, IFN-α has not demonstrated efficacy in most patients with renal cell carcinoma, although responses were seen in patients with a prior nephrectomy [27]. Although the combination of IFN-α with bevacizumab is FDA-approved, the added toxicity associated with IFN administration and the lack of evidence that the combination was superior to monotherapeutic bevacizumab has led to diminished use in clinical practice [28,29]. More recent strategies have been to combine IFN-α with other immune modulators such as anti-CTLA-4, but others such as PD-1 and PD-L1 are also ongoing [30]. 

One of the first clinically evaluated cytokines for cancer was IL-2, delivered in cationic lipids as a plasmid, and integrated into an adenovirus [31]. Glioblastoma patients were intratumorally injected with a retroviral vector carrying the IL-2 gene and the thymidine kinase gene of herpes simplex virus type 1 (HSV-TK). The HSV-TK component induces cell death, and the IL-2 gene was added to enhance the antitumor effects. In the clinical study, four patients were treated with two possible disease stabilizations [32]. In another phase I study in which a plasmid was used as the vector of the IL-2 gene, head and neck patients were intratumorally injected with the drug, which was well tolerated [33]. A similar strategy was also used in a Phase I study of prostate patients, which show an increase in the infiltration of T cells [34]. Similar findings were found when prostate patients were treated with the IL-2 gene carried by an adenovirus vector [35]. In 31 metastatic renal cell patients, a plasmid containing the gene for IL-2 had an overall response rate of 10%, 23% of patients achieved stable disease, and the median overall survival was 11 months [36]. Cumulatively, these levels of clinical activity were not sufficient to advance into later-stage clinical studies. 

More recently, attention has been directed to IL-12 as the focus for gene therapy studies given its ability to activate a variety of cytotoxic immune cells, including T cells, macrophages, and natural killer cells. In a phase I study of the IL-12 gene delivered to patients with high-grade gliomas, the production of IL-12 was controlled by the oral activator veledimex. The safety profile was acceptable, and the median overall survival was 12.7 months [8]. To further enhance activity, a trial of the combinatorial of IL-12 with anti-PD-1 has been completed, but the outcome results have not yet been released (NCT04006119) [37]. IL-12 gene therapy is also being evaluated with chemotherapy and pembrolizumab for the treatment of triple-negative breast cancer (NCT04095689). 

TNF-α has been used in cancer clinical trials for decades and in a variety of contexts, including antibody–drug conjugates, cell therapy, and fusion proteins. In a phase II study of TNF-α in combination with chemoradiotherapy in patients with locally advanced esophageal cancer, the median overall survival was 48.7 months, and the five-year survival rate was 41% [38]. TNF-α strategies have also been evaluated in patients with melanoma, rectal, pancreatic, prostate, and head and neck cancers. However, in a phase III study of pancreatic patients, there was no significant increase in overall survival [39]. Now combinations of chemokines such as TNF-α and IL-2 are being evaluated (NCT04217473).

Because chemokines regulate the migration of immune cells, therapeutic strategies that modulate such migration are now beginning to emerge [40]. For example, the gene of C-C motif chemokine ligand 21 (CCL-21) was introduced into dendritic cells and then administered to patients as an anti-tumor vaccine. In a phase I trial of patients with non-small-cell lung cancer, tumor-specific immune responses and CD8 T cell infiltration were generated [41]. Similarly, we have deposited genetically modified dendritic cells elaborating the T cell chemokine CXCL10 into the TME of preclinical glioblastoma models that enhanced survival [42]. 

## 4. TGF-B Blockade Strategies

There have been extensive efforts to target TGF-β, starting with an anti-sense gene-modified allogeneic tumor cell vaccine in lung cancer [43]. In late-stage patients, there was a 15% partial response rate, and there were increases in IFN-γ and antibody responses in clinical responders. A TGF-β anti-sense oligodeoxynucleotide AP 12,009 was noted to induce longer-than-anticipated tumor remissions in high-grade glioma patients [44]. However, later-stage clinical trials of 145 high-grade glioma patients indicated that survival and response rates were not significantly different relative to the standard of care [45]. Subsequent targeting strategies have been directed to activin receptor-like kinase (ALK1), a subclass of the TGF-β receptor, but these failed to demonstrate signals of response in urothelial cancer [46]. A strategy in which an ALK1 receptor fusion protein acts as a ligand to block signaling showed signals of response in heavily pretreated head and neck cancer, but later trials in ovarian cancer and renal cell carcinoma showed no compelling efficacy [47,48,49]. An antibody that neutralized the isoforms of TGF-β was associated with reversible cutaneous keratoacanthomas and hyperkeratosis [50] and has not been further developed. A small molecule inhibitor, LY2157299 (galunisertib), evaluated in 65 cancer patients enriched for high-grade glioma patients initially demonstrated signals of response and was without significant toxicity [51]; however, later-stage trials once again failed to show clinical benefit in randomized studies of 158 patients with glioblastoma [52]. This drug has been discontinued despite several other clinical studies attempting to use this in combination with a variety of chemotherapy and immunotherapy regimens [53,54,55]. More recent attempts at targeting TGF-β include a bifunctional fusion protein composed of the extracellular domain of the TGFβRII receptor (a TGFβ “trap”) fused to a human antibody against programmed death ligand 1 (PD-L1). In early-stage clinical trials of gastric cancer, there were signals of clinical response that correlated with TGF-β levels [56]. This therapeutic has also been evaluated in the indications of biliary cancer, head and neck cancer, papillomavirus-associated malignancies, and esophageal cancer [57,58,59,60]. A press release from Merck in 2021 indicated that they were discontinuing a late-stage clinical trial of this agent across various cancer lineages, in which TGF-β was presumed to play a role since the study was unlikely to meet the primary endpoint of progression-free survival. Nonetheless, there are active clinical studies of Bintrafusp alfa in sarcoma, neuroblastoma, thymoma, cholangiocarcinoma, HPV-associated malignancies, and genitourinary malignancies, including some that are in combination with radiation. Because there is no CLIA assay or defined cut-point for TGF-β expression, none of these studies are stratified based on this parameter.

## 5. Considerations for Specific Cytokine Targeting in Different Malignancies

There are several considerations for the selection of a therapeutic cytokine. For those instances in which an immunosuppressive cytokine is being targeted for elimination, key features for consideration include (1) expression of the target across the TME; (2) expression retained at recurrence; and (3) incidence of expression in the specific malignancy. If there is marked heterogeneity, then a companion biomarker needs to be considered. The redundancy of other mechanisms of non-targeted immune suppression and heterogeneous expression of the target within the TME or between subjects may account in part for the failure to see biological responses in some of the clinical trials. If the target is only present in a small subset of cells, then a reduction in the target would not be expected to have a meaningful clinical impact. Another confounder is the redundancy of immunosuppressive pathways, as illustrated in the case of gliomas, where there is the simultaneous expression of IL-10, VEGF, and TGF-β. Rarely is the minimal level of expression defined in preclinical models to advise the cut-point for subsequent clinical trials. Since most clinical trials are initiated in the setting of standard-of-care failure and tumor recurrence, target expression in the setting of recurrence also needs to be considered. 

## 6. Overview of This Special Issue

Because of the additional challenges posed by brain tumors, such as the blood–brain barrier (BBB), several innovative strategies such as BBB opening ultrasound may provide new avenues for overcoming some of the prior barriers [42,61]. To illuminate the evolution of chemokines and to identify new opportunities, we have commissioned review articles to stimulate scientific discourse on this topic. In the “History of Cytokine and Immune Therapy in Glioblastoma”, a more in-depth review highlights the use of cytokine-elaborating viruses administered directly into the glioblastoma TME, which minimizes the systemic toxicities commonly associated with cytokine immunotherapy. Cytokines have conventionally been used with and for the activation of a wide variety of adoptive immune therapeutic strategies, such as initially lymphocytes and NK cells and then chimeric antigen T cells. Now, genetic engineering strategies are emerging in which these cytokines are stably expressed, or immunosuppressive cytokine receptors have been knocked out to maintain their effector functions in a hostile immunosuppressive microenvironment, as reviewed in “Cytokine Modification of Adoptive Chimeric Antigen Receptor Immunotherapy for Glioblastoma”. Because the oncology field is now evolving to molecular characterization and precision-targeted strategies, there are new and likely more appropriate opportunities for patient selection, including in younger patients with high degrees of baseline immune reactivity or based on molecular drivers, as described in “Immunobiology and Cytokine Modulation of the Pediatric Brain Tumor Microenvironment: A scoping Review”. Targets such as Schlafens that have dual activities for inducing immune sensitization while simultaneously reducing cancer cell proliferation, differentiation, and invasion may be a multi-prong strategy that has a therapeutic impact, as explained in “Schlafens as Targets in the Treatment of Malignancies”.
